# Fungus Hæmatodes of the Thigh, Said to Be Cured by Amputation

**Published:** 1831

**Authors:** 


### VIII.

### FUNGUS HÆMATODES OF THE THIGH, SAID TO BE CURED BY AMPUTATION.

IN the same number of the Glasgow Journal is related a case of this description. A lad, about 23 years old, applied to Mr. M'Dowall in September 1829, with a soft swelling of the thigh from the knee to near the groin; it looked like deep-seated abscess, was punctured, but only a white fatty substance and a little blood escaped. The disease had commenced nine years previously in the form of abscess which had broken and discharged much matter. From the opening now made in the integuments, a large white fungous tumour appeared, and continued to advance. The opening being enlarged it soon attained a considerable bulk, looking like a large melon, and blood beginning to ooze from it, as if pressed from a sponge. The bleeding was troublesome, and the top began to mortify, so the tumour was removed down to the thigh-bone, and was found to penetrate deeply. The tumour, when cut into, looked soft and pulpy like brain. There was much loss of blood in the operation, and the patient grew rather collapsed. The wound appeared to be doing well, and so did the boy, but five fungous tumours soon began to appear through the wound. Every day they enlarged, and now a sharp point of bone was felt just over the head of the fibula, separate from the inside of the thigh-bone; it was removed, and another large fungous tumour, bleeding profusely, began to advance. The patient went on in a most miserable way, till the 10th December, when amputation of the thigh was performed, about four inches below the trochanter major. The health after this improved, and on the 10th April, 1830, Mr. M'Dowall met him on the road.

On examining the limb it was found that the piece of bone, measuring five inches in length, which was cut out near the head of the fibula, had been detached from the femur, and had left the cavity of that bone open to the marrow. From this opening the fungus hæmatodes had proceeded. The whole of the muscles near the knee-joint were altered in texture, and the inner side of the femur was carious for some distance above the knee.

The operation was performed in December 1829, the young man was seen well in April 1830, and the case is dated December 1830. Now if it was really one of fungus hæmatodes, and it looks extremely like it, we contend that Mr. M'Dowall is hardly justified in pronouncing that amputation has effected a cure. It is notorious that patients do often recover after operations for scirrhus and fungus hæmatodes when their situation seemed utterly desperate. They recover for a time, but seldom does this deceptive benefit prove permanent. After a variable period, it may be months or even a few years, the disease returns in the situation of the wound, in the stump, or in some other organ, cuts off the patient, and gives the lie to the chronicler of cures. The profession in general are not sufficiently aware of this circumstance, and it is only the surgeons connected with large hospitals who have an opportunity of ascertaining the results of these operations. We must hold up our hands against the system of publishing such results as determined, before a sufficient length of time can have elapsed to fix the seal of truth upon the narrative. It is a system fraught with error and delusion, calculated to mislead those who have not extensive experience of their own, entailing on the world unnecessary, cruel, and fatal operations, and offering an obstacle of the most insuperable kind to the progress of medical science. We repeat that we hold up our hands against it, and shall visit with unsparing criticism whatever instances of this most serious fault may fall beneath our notice.

